# When to suspect contamination rather than colonization – lessons from a putative fetal sheep microbiome

**DOI:** 10.1080/19490976.2021.2005751

**Published:** 2021-12-20

**Authors:** Simone Bihl, Marcus de Goffau, Daniel Podlesny, Nicola Segata, Fergus Shanahan, Jens Walter, W. Florian Fricke

**Affiliations:** a Department of Microbiome Research and Applied Bioinformatics, University of Hohenheim, Stuttgart, Germany; b Department of Vascular Medicine, Academic Medical Center, University of Amsterdam, Amsterdam, The Netherlands; c Wellcome Trust Sanger Institute, Wellcome Trust Genome Campus, Cambridge, UK; d Department CIBIO, University of Trento, Trento, Italy; e APC Microbiome Ireland, Department of Medicine, University College Cork, Ireland; f Institute for Genome Sciences, University of Maryland School of Medicine, Baltimore, MD, USA

**Keywords:** Fetal microbiome, sheep, contamination, metagenomics, metatranscriptomics, microbial strains, phiX

## Abstract

There is an ongoing controversy around the existence of a prenatal, fetal microbiome in humans, livestock, and other animals. The ‘*in utero* microbial colonization’ hypothesis challenges the clinical paradigm of the ‘sterile womb’ but has been criticized for its reliance on DNA-based evidence to detect microbiomes and the failure to conciliate the routine experimental derivation of germ-free animals from surgically resected embryos with a thriving fetal microbiome. In order to avoid the propagation of misinformation in the scientific literature, a critical assessment and careful review of newly published studies, particularly those that challenge the convincing current clinical dogma of the sterile womb, is of critical importance.

We read with interest a recent publication that postulated the presence of a fetal microbiome in sheep, but questioned the plausibility of the reported findings and their meaningfulness to prove “microbial colonisation of the fetal gut […] *in utero*”. We reanalyzed the published metagenomic and metatranscriptomic sequence data from the original publication and identified evidence for different types of contamination that affected all samples alike and could explain the reported findings without requiring the existence of a fetal microbiome.

Our reanalysis challenges the reported findings as supportive of a prenatal fetal lamb microbiome. The shortcomings of the original analysis and data interpretation highlight common problems of low-biomass microbiome projects. We propose genomic independence of separate biological samples, i.e. distinctive profiles at the microbial strain level, as a potential new microbiome marker to increase confidence in metagenomics analyses of controversial low-biomass microbiomes.

The scientific community continues to debate whether there exists a prenatal, fetal microbiome in humans and related animals. At this advanced stage of the controversy, the most important arguments from both sides of the debate have been laid out,^1,2^ and impartial experts have weighed in on some of the underlying problems of the discussion, such as the definition of what constitutes a microbiome and what type of experimental support is needed to prove its existence.^3^ The philosophical framework behind some of the supportive research of the *in utero* colonization hypothesis has been questioned,^4^ and a refocus of the debate on the clinical relevance of any type of microbial communication with the human or animal host during pregnancy has been proposed.^5^ Meanwhile, new studies continue to be published in favor^6^ or against^7^ the fetal microbiome hypothesis.

In this situation, the scientific community bears an increased responsibility to scrutinize new findings, manuscripts, and publications. There is a critical need for microbiome researchers to carefully question their findings before publication and for an impartial, competent assessment of new manuscripts by the journals at the editorial level, by expert scientists during peer review and by the broader scientific community after publication. The following example of a problematic study in favor of the fetal microbiome hypothesis highlights the dangers of misinterpreting erroneous microbiome data resulting from contamination to provide unjustified arguments for one side of a highly controversial scientific debate.

In the study “Multiomics analysis reveals the presence of a microbiome in the gut of fetal lambs”, Bi *et al*. applied metagenomics, metatranscriptomics, metabolomics, and real-time PCR (qPCR) to study the cecal microbiota of fetal lambs after C-section and reported “strong evidence that the prenatal gut harbours a microbiome and that microbial colonization of the fetal gut commences *in utero*”.^8^ Their claim is surprising because lambs can be raised germ-free after hysterectomy,^9^ which argues against the presence of live bacteria in the fetus before birth. Microbial colonization was also not experimentally verified, as the authors did not attempt bacterial cultivation as evidence for *in utero* colonization – surprisingly, as *Escherichia coli*, which was detected as the dominant bacterium in all fetal samples, is easily cultivable and unlikely to escape cultivation attempts. We reanalyzed the metagenomic and metatranscriptomic data that were made available and identified homogenous and concerning metagenome and metatranscriptome sequence compositions that are more consistent with massive contamination than with a fetal lamb microbiome.

In the original paper, the authors isolated DNA and RNA from the cecal contents of six healthy lambs (C1-C6) delivered by aseptic C-section, as well as a negative control consisting of nucleic acid-free water, all of which were subjected to metagenomic and metatranscriptomic sequencing on the Illumina HiSeq platform. The published data also contains a positive control, which is not described in the paper. After quality control (adapter trimming and read filtering based on base call quality) and read mapping to the sheep (*Ovis aries*) genome, the authors were left with 10,544,549 metagenomic reads (1,757,425  ±  562,944 reads per sample) and 56,746,269 metatranscriptomic reads (9,457,711  ±  5,982,821 reads per sample). For taxonomic analysis, a gene-centric, assembly-based approach was used that resulted in a total of 19,320 and 1,691 non-redundant genes in the combined metagenomes and metatranscriptomes, respectively. To identify contamination, the relative abundances of genes were compared between samples and negative controls, based on the number of individual reads that could be mapped back to gene calls. Only genes with a ‘log2FoldChange>2ʹ in relative abundance (i.e. with a >4-fold higher mean relative abundance in samples compared to the negative control) were kept for downstream analysis, although the details of the relative gene abundance calculation are not clear and not described in the publication. Contaminant removal reduced the number of non-redundant genes available for downstream analyses to 14,199 and 1,456 for metagenomes and metatranscriptomes, respectively. Taxonomic profiles were estimated based on BLASTx comparisons of translated gene sequences to the non-redundant protein database at NCBI, which were summarized at the phylum, genus, and species level.

The authors quantified ‘the copy numbers of total bacteria’ as 4.6 × 10^7^  ±  3.4 × 10^7^ and 1.6 × 10^7^  ±  1.1 × 10^6^ in cecal content samples and negative controls, respectively, based on qPCR. They identified a microbiota of low ɑ-diversity dominated by the phylum *Proteobacteria* (95.30% ± 2.19%), with additional presence of *Firmicutes* (4.85% ± 1.71%), *Actinobacteria* (0.53% ± 0.22%) and *Thaumarchaeota* (0.02% ± 0.01%). *Escherichia coli* (86.89% ± 2.21%) and *Catellicoccus marimammalium* (4.11% ± 1.61%) were identified as the dominant bacterial species. The authors also identified bacteriophage *phiX174* (52.29% ± 5.55% of all mapped reads) and Orf virus (0.03% ± 0.01% of all mapped reads) in the samples, although again, it is unclear how the relative abundances of bacteria and viruses were calculated.

For our reanalysis, we applied a straightforward and efficient approach to identify and compare major host and microbial components in the raw, published metagenomes (PRJNA601636) and metatranscriptomes (PRJNA598075). After a quality filtering with KneadData v0.6.1 (https://huttenhower.sph.harvard.edu/kneaddata) that was similar to the method used by the authors (SLIDINGWINDOW:4:20, MINLEN:70; i.e. trimming of sequence regions with a base call quality below Q20 and removal of reads that were truncated by >30%), reads were mapped to eukaryotic, bacterial, and viral reference genomes with the Burrows-Wheeler Aligner,^10^ using default parameters. As sequence similarities between genomes from distinct taxa can result in the cross-mapping of reads that confound taxonomic assignments and relative abundance estimates, we used an iterative read mapping and removal method to identify host and microbial DNA and RNA contributions to the sequence data. Importantly, our metagenome and metatranscriptome reanalysis points to different types of contamination that affected all samples and controls alike, i.e. independently of sample-specific relative abundance estimates, raising doubts about any claims of a fetal lamb microbiota from the original publication. In the following, our concerns are described in detail.

## 1. Sample (cross-)contamination

**Figure 1.**
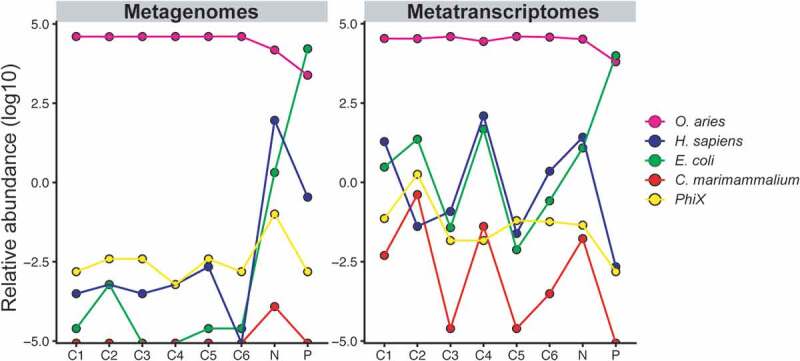
Comparable relative abundance profiles in different DNA and RNA samples and controls. Relative abundances were calculated based on the number of reads mapped to reference genomes with BWA, either relative to the total number of quality-filtered reads (for *O. aries*) or relative to the number of qualify-filtered reads after removal of sheep sequence data (all others). Reads were mapped iteratively using a filtering approach, i.e. only those reads that did not map to one genome were used as input for the mapping to the next genome, in the order *O. aries* -> *H. sapiens* -> *E. coli* -> *C. marimammalium* -> *phiX.*

In agreement with the original publication, our taxonomic profiling identified eukaryotic (sheep and human), bacterial (*E. coli* and *C. marimammalium*), and viral (*phiX174*) DNA and RNA fractions in all samples (Table 1). We did not detect Orf virus after filtering out reads that mapped to the sheep genome and mapped reads of the unfiltered data covered only ~10% of the Orf genome, suggesting a misclassification of virus-related sheep genome sequences as Orf virus in the original publication. More importantly, the same eukaryotic, bacterial, and viral species were detected in all 16 samples, i.e. distinct metagenomes and metranscriptomes from each of the six fetal lamb samples (C1-6), as well as the positive (P), and negative (N) controls (Table 1). Moreover, the identified bacterial and viral species were detected in similar proportions in all fetal lamb samples and at comparable or even higher relative abundance in the negative control (Figure 1). According to the cited contamination removal procedure, all of the reported microbial sequence fractions should have been excluded from the analysis based on overlapping microbiome profiles with the negative control. Well-to-well cross-contamination,^11^ contamination from extraction kits^12^ or other sources in the lab environment are known to disproportionately affect low-biomass microbiome samples.^13^ Our reanalysis thus suggests that the major bacterial and viral members of the proposed fetal lamb microbiota can be attributed to background signals from sample contamination – reminiscent of similar findings from the analysis of human placenta samples,^14^

**Table 1.** Overview of the original sequence data and the results of their re-analysis

Sample/template		Low-quality and host DNA removal Host DNA removal				Taxonomic analysis [reads/% of processed and host DNA-filtered]			
		Raw sequence data^1^ [reads]	Low quality read filtering^2^ [reads/% of total]	*O. aries* (sheep)^3^ [reads/% removed]	Filtered sequence data [reads/% of total]	*H. sapiens*^4^ [reads/% of filtered]	*E. coli*^5^ [reads/% of filtered]	*C. marimammalium*^6^ [reads/% of filtered]	Phi X^7^ [reads/% of filtered]
C1	DNA	94,721,122	7,504,441/7.9%	87,048,840/99.8%	167,841 / 0.2%	25,670/15.3%	4,792/2.9%	25/<0.1%	48,122/28.7%
	RNA	8,554,736	1,326,647/15.5%	6,741,078/93.3%	487,011/5.7%	261,609/53.7%	117,237/24.1%	7,375/1.5%	23,255/4.8%
C2	DNA	106,325,910	7,925,261/7.4%	97,731,410/99.3%	669,239/0.6%	38,358/5.7%	39,768/5.9%	1,157/0.2%	87,724/13.1%
	RNA	8,153,304	1,258,545/15.4%	6,392,044/92.7%	502,715/6.2%	17,564/3.5%	268,923/53.5%	46,912/9.3%	89,231/17.7%
C3	DNA	110,048,306	100,991,077// 91.8%	100,761,731/99.8%	229,346/0.2%	29,312/12.8%	4,835/2.1%	16/<0.1%	93,480/40.8%
	RNA	39,103,292	33,979,905/86.9%	33,645,902/99.0%	334,003 / 0.9%	135,772/40.7%	81,504/24.4%	3,353/1.0%	54,540/16.3%
C4	DNA	95,215,914	87,445,966/91.8%	87,230,854/99.8%	215,112/0.2%	36,214/16.8%	4,226/2.0%	56/<0.1%	36,821/17.1%
	RNA	8,656,196	7,313,727/84.5%	6,210,900/84.9%	1,102,827/12.7%	594,911/53.9%	391,563/35.5%	18,067/1.6%	11,739/1.1%
C5	DNA	117,291,656	107,795,471/91.9%	107,520,176/99.7%	275,112/0.2%	75,921/27.6%	5,838 /2.1%	19/<0.1%	96,498/35.1%
	RNA	30,009,606	26,535,222/88.4%	26,345,106/99.3%	190,116/0.6%	54,327/28.6%	30,887/16.2%	1,586/0.8%	80,009/42.1%
C6	DNA	149,365,318	137,894,749/92.3%	137,772,043/99.9%	122,706/0.1%	6,872/5.6%	9,435/7.7%	72/0.1%	89,478/72.9%
	RNA	19,015,404	16,674,129/87.7%	16,219,742/97.3%	454,387/2.4%	236,101/52.0%	92,953/20.5%	5,045/1.1%	47,720/10.5%
Negative control	DNA	4,956,844	429,994/8.7%	2,953,335/65.2%	1,573,515/31.7%	321,548/20.4%	62,139/3.9%	1,129/<0.1%	16,879/1.1%
	RNA	7,707,198	1,283,593/6.7%	5,866,837/91.3%	556,768/7.2%	266,483/47.9%	189,912/34.1%	11,187/2.0%	16,819/3.0%
Positive control	DNA	118,227,748	8,647,781/7.3%	32,281,742/29.5%	77,298,225// 65.4%	690,722/0.9%	74,205,991 / 96.0%	3,128/<0.1%	69,080/0.1%
	RNA	51,212,242	3,641,662/7.1%	21,274,220/44.7%	26,296,360/51.3%	34,128/0.1%	25,793,455/98.1%	877/<0.1%	26,780/0.1%

^1^Raw sequence data from the original manuscript were downloaded from https://www.ncbi.nlm.nih.gov/sra/ (PRJNA601636; PRJNA598075);

^2^Sequence regions where the base quality fell below Q20 within a 4-nucleotide sliding window were trimmed and reads that were truncated by more than 30% removed (SLIDINGWINDOW:4:20, MINLEN:70) with KneadData v0.6.1 (https://huttenhower.sph.harvard.edu/kneaddata);

^3^Host DNA was removed by mapping trimmed reads to the sheep genome (*Ovis aries*; GCF_002742125.1) with the Burrows-Wheeler Aligner (BWA; http://bio-bwa.sourceforge.net/);

^4−9^For taxonomic assignments, reads were successively mapped to the different eukaryotic, bacterial, and viral reference genomes with BWA. After each alignment, mapped reads were filtered out and only the remaining reads compared to the next reference, in the order *O. aries* -> *H. sapiens* -> *E. coli* -> *C. marimammalium* -> *phiX*.

^4^*Homo sapiens* ((GRCh37/hg19));

^5^*Escherichia coli* K12 DH10B (GCF_000019425.1);

^6^*Catellicoccus marimammalium* M35/04/3 (GCF_000313915.1);

^7^*Escherichia virus phiX174* (GCF_000819615.1).

## 2. Conspicuous, potentially lab-derived bacteria and viruses

*E. coli* is an easily cultivable, ubiquitous prokaryotic model species, which is found in human, animal, and environmental samples^15^ and is frequently used as a lab strain. It is thereforelikely that Bi et al. used DNA and RNA from an *E. coli* lab strain as the positive controls for metagenomic and metatranscriptomic sequencing. This is suggested by the dominance of reads (>95% of reads after filtering for low quality, sheep and human DNA) that could be mapped to the *E. coli* genome (Table 1). The second most abundant bacterial species, *C. marimammalium*, a member of the gull fecal microbiota,^16^ has been identified in seal and porpoise^17^ and is used as a marker for gull-associated fecal contamination.^18^ To our knowledge, it has never been identified in land animals. Bacteriophage *phiX174* is a model virus infecting *E. coli* and used as an internal, spike-in DNA sequencing control, including on the Illumina platform used by Bi et al. Raw data preprocessing before analysis typically involves phiX sequence removal, which, if inadequately performed, can result in phiX-contaminated sequence data that have been frequently documented for microbial isolate genome sequences.^19^ In summary, the most abundant bacterial and viral species in the sheep (and control) samples would seem unlikely to have colonized the fetal lamb, escape cultivation-based detection or allow for the generation of germ-free sheep. They could, however, have easily been transferred as contaminants from other, unrelated samples, sequencing projects, or laboratory reagents. Without further experimental support, these microbes do not provide convincing arguments for the presence of a fetal lamb microbiota.

## 3. Identical E. coli and PhiX strains in independent samples and controls

**Figure 2.**
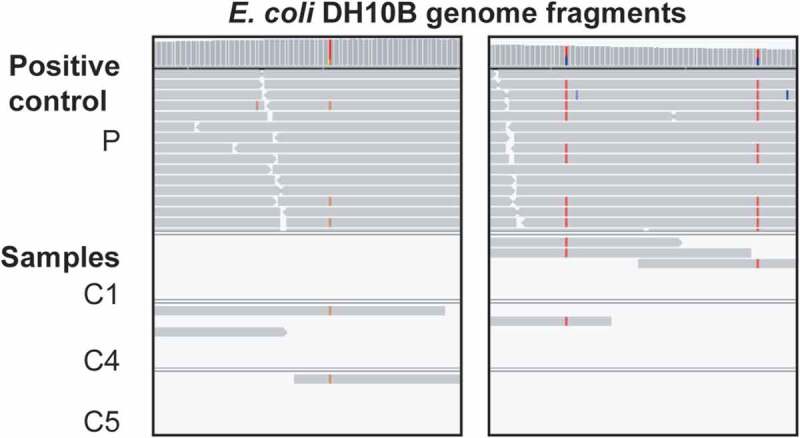
Shared single-nucleotide variants in fetal lamb metagenomes and positive control identify the same strain. Two screenshots show trimmed and filtered metagenomic reads mapped with BWA to *E. coli* K12 DH10B (GCF_000019425.1). Alignments were visualized with the Integrative Genomic Viewer (IGV; https://software.broadinstitute.org/software/igv/). Based on unique and shared single nucleotide variation (SNV) profiles the positive control (p) contains two strains. With respect to the depicted genome region, one strain is identical to DH10B whereas the other strain is also found in three fetal lamb samples (C1, C4, C5)

**Figure 3.**
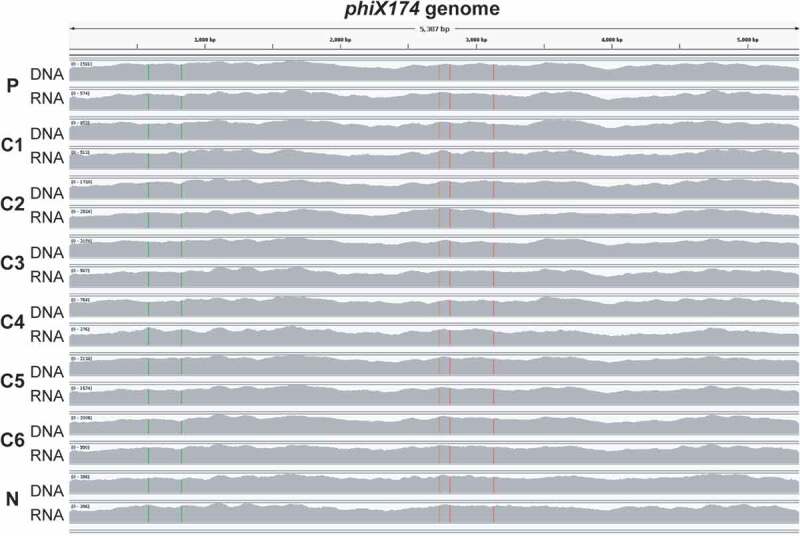
Sample and control metagenomes and metatranscriptomes contain the same *phiX* strain. A screenshot shows trimmed and filtered metagenomic and metatranscriptomic reads mapped with BWA to *phiX174*(GCF_000819615.1). Sequencing coverage in all datasets extends over the entire phage genome (100%), including non-transcribed regions around the origin of replication

Reads from the positive control showed 100% average nucleotide identity (fastANI^20^) to the genome of *E. coli* K12 DH10B (less for *E. coli* K12 MG1655), which is commercially available from Invitrogen (RefSeq: GCF_000019425.1), suggesting that this lab strain was used as the positive control. Shotgun metagenomics can provide taxonomic resolution down to the level of individual microbial genomes, allowing for the differentiation of even closely related individual strains with distinct single-nucleotide variant (SNV) profiles,^21^ such as distinct *E. coli* isolates.^22^ We detected strain-specific SNV profiles in the alignment of mapped reads from the positive control to the *E. coli* K12 DH10B reference genome and identified two distinct strains, one of which could also be detected in several fetal lamb metagenomes, based on shared SNVs in overlapping mapped read sections (Figure 2). The substantially higher sequencing depth of *phiX174* in all samples allowed for an even more unequivocal strain detection based on shared SNV profiles and identified the same viral strain in all metagenomes and metatranscriptomes, including positive and negative controls (Figure 3). As identical bacterial and viral strains would be less likely to naturally occur in independent biological samples than to result from well-to-well contamination,^11^ the detection of shared strains in fetal lamb samples and controls provides additional evidence in favor of metagenomic and metatranscriptomic data contamination and against the presence of a fetal lamb microbiome.

## 4. DNA contamination of metatranscriptome sequencing templates

The isolation of RNA from microbiome samples is prone to contamination with traces of metagenomic DNA and requires extensive DNase treatment,^23^ Our reanalysis shows that mapped metatranscriptome reads from all fetal lamb samples, including positive and negative controls, span the entire *phiX174* genome (Figure 3). This includes genome regions that have been shown to be non-transcribed, such as around the origin of replication.^24^ Our findings therefore indicate that at least a fraction of the metagenomic sequence data must have been derived from DNA templates, providing strong evidence for an additional source of contamination in the fetal lamb metatranscriptomes, which refutes Bi et al.’s claim of “support that the microbiome(s) present in the prenatal fetal gut are active”.

Collectively, our analysis suggests that contamination and technical problems account for at least substantial fractions if not all of the microbiome-associated findings from Bi *et al*.: Sheep, human and microbial DNA and RNA in positive and negative controls, identical *E. coli* and *phiX174* strains in independent samples and controls, and DNA-derived signals in metatranscriptomes, all point to massive external and/or internal contaminations from other samples, reagents or the lab environment. Contamination is a well-known problem for sequencing-based microbiota studies and careful experimental and bioinformatic measures have been proposed to thoroughly assess and reduce its impact on low-biomass microbiome studies.^25^ It has therefore been argued repeatedly that the identification of metagenomic DNA in a sample is insufficient to postulate the presence of a microbiome.^3^ Metatranscriptomic RNA indicates transcriptional activity but has to be carefully controlled for contaminating DNA^23^ and may be similarly influenced by contamination as metagenomic DNA.^26^ The isolation of cultivable bacteria therefore remains the strongest evidence for a physiologically active microbiome. Why this experimental evidence of a fetal lamb microbiome was not requested during peer review of the paper by Bi et al is incomprehensible, especially with regard to supposedly abundant (‘88.76% ± 2.04%’ of ‘4.6 × 10^7^ [‘copy numbers per gram of total bacteria in cecal content samples’]) and easily cultivable *E. coli* strains detected in the samples.

We would like to emphasize that our data reanalysis suggests the utility of another control for controversial microbiome studies, which has not yet received much attention in the field, i.e. the proof of biological independence of distinct microbiome samples. Unless microbiome samples originate from very related individuals, such as for example animal litter mates, neonates, and their mothers,^27^ or recipients of a fecal transplantation and their donors,^28^ their microbiomes should contain genomically distinct microbial strains, even within the same species. Metagenomics provides the phylogenetic resolution to differentiate between these related strains, particularly with sufficient sequencing depth,^28,29^ but as our reanalysis demonstrates, also for more shallow sequence data from low-biomass samples.^30^

Science is said to be self-correcting when others reproduce or refute published findings. We call on leading journals in providing more critical reviews (including our contribution), particularly when claims are obviously contentious^5^ or in conflict with stronger experimental evidence (e.g. the derivation of germ-free lambs).^4^

## Data Availability

The manuscript presents a reanalysis of previously released sequence data.
